# Common Variable Immunodeficiency patients with a phenotypic profile of immunosenescence present with thrombocytopenia

**DOI:** 10.1038/srep39710

**Published:** 2017-01-05

**Authors:** Jan Stuchlý, Veronika Kanderová, Marcela Vlková, Ivana Heřmanová, Lucie Slámová, Ondřej Pelák, Eli Taraldsrud, Dalibor Jílek, Pavlína Králíčková, Børre Fevang, Marie Trková, Ondřej Hrušák, Eva Froňková, Anna Šedivá, Jiří Litzman, Tomáš Kalina

**Affiliations:** 1 CLIP - Childhood Leukemia Investigation Prague, Department of Paediatric Haematology and Oncology, Second Faculty of Medicine, Charles University and University Hospital Motol, Prague, Czech Republic; 2 Department of Clinical Immunology and Allergology, St. Anne’s University Hospital, Brno, Czech Republic; 3 Faculty of Medicine, Masaryk University, Brno, Czech Republic; 4 Department of Cancer Immunology, Institute for Cancer Research, Oslo University Hospital Radiumhospitalet, Oslo, Norway; 5 Centre of Immunology and Microbiology, Regional Institute of Public Health, Usti nad Labem, Czech Republic; 6 Institute of Clinical Immunology and Allergology, University Hospital, Hradec Kralove, Czech Republic; 7 Research Institute of Internal Medicine, Oslo University Hospital, Rikshospitalet, Oslo, Norway; 8 Section of Clinical Immunology and Infectious Diseases, Oslo University Hospital, Rikshospitalet, Oslo, Norway; 9 Gennet, Prague, Czech Republic; 10 Department of Immunology, Second Faculty of Medicine, Charles University and University Hospital Motol, Prague, Czech Republic

## Abstract

Common variable immunodeficiency (CVID) is a heterogeneous group of diseases. Our aim was to define sub-groups of CVID patients with similar phenotypes and clinical characteristics. Using eight-color flow cytometry, we analyzed both B- and T-cell phenotypes in a cohort of 88 CVID patients and 48 healthy donors. A hierarchical clustering of probability binning “bins” yielded a separate cluster of 22 CVID patients with an abnormal phenotype. We showed coordinated proportional changes in naïve CD4+ T-cells (decreased), intermediate CD27− CD28+ CD4+ T-cells (increased) and CD21low B-cells (increased) that were stable for over three years. Moreover, the lymphocytes’ immunophenotype in this patient cluster exhibited features of profound immunosenescence and chronic activation. Thrombocytopenia was only found in this cluster (36% of cases, manifested as Immune Thrombocytopenia (ITP) or Evans syndrome). Clinical complications more frequently found in these patients include lung fibrosis (in 59% of cases) and bronchiectasis (55%). The degree of severity of these symptoms corresponded to more deviation from normal levels with respect to CD21low B-cells, naïve CD4+ and CD27− CD28+ over three years. Moreover, th-cells. Next-generation sequencing did not reveal any common genetic background. We delineate a subgroup of CVID patients with activated and immunosenescent immunophenotype of lymphocytes and distinct set of clinical complications without common genetic background.

Common variable immunodeficiency (CVID) is a heterogeneous collection of diseases defined as hypogammaglobulinemia of unknown cause (secondary hypogammaglobulinemia excluded) with markedly decreased IgG and IgA levels, with or without low IgM levels, displaying a lack of antibody response to vaccination. It is clinically accompanied by infections, autoimmunity, granulomatous disease and, in some cases, lymphoproliferation. In a large study by Resnick^1^, 94% of the patients had a history of infections, while autoimmunity was found in 28% of the patients which is similar to 29% reported by European Society for Immunodeficiencies Registry Working Party^2^. The most frequent autoimmune condition was immune thrombocytopenia (14%). Interestingly, 32% of the patients were affected by infections only and had significantly increased survival compared to patients with other complications. The heterogeneity in clinical presentation, the relative rarity of the disease and the thus far elusive molecular pathogenesis are factors inhibiting progress in understanding the disease and the development of better therapeutic approaches. Abnormalities have predominantly been found in the phenotype of B-cells, leading to the development of several classification schemes (Paris^3^, Freiburg^4^, EUROClass^5^, Rotterdam^6^), but other reports describe changes in the T-cell compartment as well^7^,^8^,^9^,^10^. Within B-cell abnormalities, immunophenotyping by flow cytometry shows a lack of switched memory B-cells and increased levels of transitional B-cells and CD21low B-cells^11^. While the lack of switched memory B-cells is consistent with the failure to produce antibodies in germinal centers, the increased levels of transitional B-cells (with poor regulatory function in CVID) may be a result of T-cell activation^12^. CD21low B-cells have been described as tissue-homing, innate-like memory cells^13^ with extensive proliferation history^13^,^14^, capable of autoreactivity^15^ but also with limited responsiveness^16^. These puzzling CD21low B-cells have been found in increased amounts in CVID patients with autoimmune cytopenias and in patients with systemic lupus erythematosus^17^, rheumatoid arthritis^15^ and Sjögren’s syndrome^16^. The abnormal phenotypic profile of B-cells in CVID is remarkably stable^18^. The numbers of CD4+ T-cells in CVID were reported to be decreased^8^, with a marked loss of naivety^8^,^9^ and lower numbers of T regulatory cells^19^.

In this study, in order to better grasp the heterogeneity of CVID, we delineated subgroups of CVID with particular phenotypic and clinical features. We used a systems biology approach to group together patients with similar B-cell and CD4+ T-cell phenotypes. Then, we aimed to define the clinical, cellular and cytokine profile of the most strikingly different subgroup of CVID.

## Materials and Methods

### Patients and healthy donors

Eighty-eight CVID patients diagnosed according to the European Society for Immunodeficiencies criteria^20^ and 48 healthy controls of Caucasian origin were enrolled in the study in the period 2010–2014 and provided written informed consent. Patient cohort included unselected patients in non-acute condition who were cared for in Prague, Brno, Ústí nad Labem, Hradec Králové and Oslo regional centers. In addition to 88 patients described here, 10 patients were excluded from the probability binning analysis as they were lacking B cells (<1% of lymphocytes). The characteristics of the patients are shown in Table 1. The study was approved by the institutional review boards of University Hospital Motol in Prague, St. Anne’s Faculty Hospital in Brno and Regional Committee for Medical and Health Research in Oslo, Norway and it was carried out in accordance with the Declaration of Helsinki and corresponding local law. Thrombocyte counts were taken from the routine hospital complete blood count evaluations in the same period as was the sample for immunophenotyping (median difference 15 days). The presence of bronchiectasis, lung fibrosis and emphysema was determined by high-resolution computerized tomography (HRCT), involvement of 1–3 lobes or >3 lobes was determined. Splenomegaly was defined as a spleen length exceeding 12 cm on ultrasonography. Lymphadenopathy was defined as the presence of palpable lymph nodes on at least two different sites of the body or increased lymph nodes as determined by ultrasonography or CT scan. All patients received intravenous or subcutaneous immunoglobulin (Ig) replacement therapy, and blood samples were always collected before the treatment.

### Cell separation and immunophenotyping by flow cytometry

Peripheral blood mononuclear cells (PBMCs) were isolated from 10 ml EDTA blood by density gradient centrifugation using a Ficoll-Paque (GE Healthcare Bio-Sciences, Uppsala, Sweden). The isolated PBMCs were washed twice with PBS supplemented with 1% (w/v) bovine serum albumin (BSA). The single cell suspension was incubated with “B cell or T cell antibody cocktail” for 15 min in the dark at room temperature. “B cell antibody cocktail” included anti-CD20 peridinin chlorophyll (PerCP), anti-CD27 Pacific Blue (PB), and anti-CD38 Alexa-Fluor 700 (Exbio Praha a.s., Vestec, Czech Republic), anti-human IgM fluorescein isothiocyanate (FITC), and anti-CD21 allophycocyanin (APC; BD Biosciences, San Jose, CA), anti-CD19 phycoerythrin-cyanin 7 (PC7), and anti-CD24 phycoerythrin (PE; Immunotech, Marseille, France) and anti-IgD biotin (SouthernBiotech, Birmingham, AL, USA) followed by streptavidin-Qdot 605 (Invitrogen, Carlsbad, CA). “T cell antibody cocktail” included anti-CD4 Alexa-Fluor 700 and anti-CD27 PB (Exbio Praha a.s.), anti-CD3 PerCP-cyanin 5.5 (PerCP-Cy5.5) and anti-CD279 APC (Affymetrix eBioscience, San Diego, CA, USA), anti-CD8 Horizon^TM^ V-500 and anti-CD45RA PC7 (BD Biosciences), anti-CD38 PE (Immunotech), and anti-CD57 biotin (BD Biosciences) followed by streptavidin-Qdot 605 (Invitrogen). After a final wash with PBS, two million PBMCs from each sample were acquired using a Cyan ADP flow cytometer (Dako, Glostrup, Denmark). For the detection of activation markers, PBMCs were isolated from 4.5 mL EDTA blood by density gradient centrifugation using a Ficoll-Paque (GE Healthcare), the isolated PBMCs were washed twice with PBS containing 1% (w/v) BSA and the single cell suspension was incubated with a mixture of anti-CD27 PB and anti-CD38 Alexa-Fluor 700 (Exbio Praha), anti-human IgM FITC, anti-CD21 APC, anti-CD24 APC-H7, and anti-CD70 PE (BD Biosciences), anti-CD19 PC7 (Immunotech), and anti-CD69 PerCP-Cy5.5 (BioLegend, San Diego, CA, USA) for the “B-lineage tube” and with a mixture of anti-CD4 Alexa-Fluor 700 and anti-CD27 PB (Exbio Praha), anti-CD3 APC-H7, anti-CD8 Horizon^TM^ V-500, anti-CD45RA PC7, and anti-CD70 PE (BD Biosciences), anti-CD28 Alexa-Fluor 488, anti-CD197 APC, and anti-CD69 PerCP-Cy5.5 (BioLegend) for the “T-lineage tube”. After a final wash, the samples were collected using LSR II flow cytometer (BD Biosciences) and analyzed using FlowJo software (Treestar, Ashland, OR, USA) or the computational tools described below.

### Flow cytometric arrays for cytokine analysis

PBMCs were separated using Ficoll-Paque gradient (GE Healthcare) and the upper plasma layer was used for the detection of cytokines. Interleukin (IL)-1b, IL-4, and IL-5 were measured using Flow Cytomix^TM^ Multiplex Technology (Affymetrix eBioscience). IL-9, granulocyte-colony stimulating factor (G-CSF), and granulocyte-macrophage colony-stimulating factor (GM-CSF) were examined using BD Cytometric Bead Array (CBA) Flex Set (BD Biosciences). IL-2, IL-6, IL-8, IL-10, IL-12, tumor necrosis factor (TNF) alpha, and interferon (IFN) gamma were examined by BD CBA Enhanced Sensitivity Flex Set (BD Biosciences). The three systems employ particles with discrete fluorescence intensities and bound antibodies to detect soluble analytes. The manufacturers’ instructions were followed. Briefly, 25 uL and 50 uL plasma sample was incubated with Flow Cytomix beads or CBA beads, respectively, and the bound analyte was labeled using a PE conjugated detection reagent and measured on the flow cytometer. LSR II flow cytometer discriminated between individual bead populations (a minimum of 300 beads in each population were acquired).The amount of bound cytokine was expressed as the median fluorescence intensity (MFI) in PE channel (576/26 nm). The concentration of a particular cytokine was calculated based on the MFIs of the range of standards supplied by the manufacturers.

### ELISA protocols

Soluble CD21 was quantified in plasma samples using Human CD21 Enzyme-linked immunosorbent assay (ELISA) according to manufacturer’s instructions (BioVendor, Brno, Czech Republic). The protocol and calibrators allowed for the detection of soluble CD21 in the range of 3.12–100 U/mL. Viral CMV IL-10 and EBV IL-10 were quantified in the plasma samples using home-made ELISA. The CMV IL-10 capture antibody (Viral HCMV IL-10 Affinity Purified Polyclonal Ab AF117, R&D Systems, Minneapolis, MN, USA) was used at 5 μg/mL, and the biotinylated detection antibody (Viral HCMV IL-10 Biotinylated Affinity Purified Polyclonal Ab BAF117, R&D Systems) at 1 μg/mL. Streptavidin-HRP at 0.5 μg/mL and ready-to-use TMB substrate (both Sigma-Aldrich, St. Louis, MO, USA) were used for the visualization. The detection range was set up using recombinant CF cmvIL-10 protein (R&D Systems) to be between 125 pg/mL and 2000 pg/mL. For EBV, the EBV IL-10 capture antibody (Viral EBV IL-10 Affinity-purified Polyclonal Goat IgG AF915, R&D Systems, MN, USA) was used at 4 μg/mL followed by the detection antibody (Viral EBV IL-10 Monoclonal Mouse IgG1 MAB9151, R&D Systems) at 2 μg/mL and secondary anti-mouse HRP-conjugated antibody (Bio-Rad, Hercules, CA, USA) at 1 μg/mL. A ready-to-use TMB substrate (Sigma Aldrich) was used for the visualization. The detection range was set up using the Recombinant CF ebvIL-10 protein (R&D Systems) to be between 62.5 ng/mL and 1000 ng/mL. The absorbance was measured at 450 nm and analyzed with VERSAmax Tunable Microplate Reader with appropriate SoftMaxPro software (Molecular Devices, Sunnyvale, CA, USA).

### Whole exome sequencing (WES) and variant filtering

DNA from PBMCs was extracted using a QIAamp DNA MiniKit (QIAGEN, Hilden, Germany). Whole exome sequencing was performed using either Nimblegen SegCap ERZ Human Exome Library v 2.0 (Roche Nimblegen, Madison, WI) or Agilent SureSelect QXT Human All Exon V5 + UTRS (Agilent Technologies, Santa Clara, CA) on HiSeq 2000 or NextSeq 500 platform (Illumina, San Diego, CA) instruments according to manufacturers’ protocols. Sequence reads were aligned against the human reference genome hg19 using BWA and postprocessed with GATK^21^,^22^. The vcf files were analyzed and filtered using Ingenuity^®^Variant Analysis™ Software (QIAGEN). First, confidence filtering was applied to exclude low quality variants and variants with low coverage (read depth at least 10). Subsequently, population databases (1000 Genomes, ExAC, NHLBI ESP) were used to remove variants with a frequency of more than 3% or 1% for Table 1A or B, respectively. Only the variants predicted as deleterious either by SIFT^23^ or Polyphen-2^24^ were further used for the analysis shown in Table 1B. The variants in genes associated with immunodeficiency were further selected using IVA biological context filtering. All listed variants were manually inspected using the Integrative Genome Viewer (IGV)^25^.

### Computational analysis

FCS files were normalized using the method based on landmark registration^26^,^27^. A probability binning algorithm was applied on the normalized dataset as described previously^18^,^28^,^29^, using flowFP implementation in R package with the following modification – the algorithm preferentially split the bins above the fluorescence background threshold (the threshold is set for each channel separately) to improve the resolution of non-negative populations. To create a reference matrix, only healthy donors’ samples were used. In short, 20,000 B cells from each healthy donor were combined together, 2048 bins containing an equal number of cells were generated by successive division. To detect populations distinguishing two sets of patient samples, a new reference probability binning model was created on dataset sampled from both sets (such that each set contributes the same number of events). Multiple testing procedures method^30^ was then used to discover differently “fed” bins with respect to this model. Classfication tree algorithm (C4.5) was used to find best subsets classifying cluster CVID-AcT. Fisher’s exact test was used to assess the significance of the frequency distribution, Wilcoxon signed-rank test was used for the comparison of metric variables between two samples, and linear regression was used for multivariate analysis. To assess the correlation of variables, the Pearson’s product-moment correlation and the Spearman’s rank correlation were used for metric and ordinal variables, respectively. P-values were adjusted using the Benjamini & Yekutieli step-up FDR-controlling procedure, and tests were considered significant if the adjusted p value was <0.05. All statistical tests and regression diagnostics were performed in R-project/Bioconductor http://www.r-project.org.

## Results

### Immunophenotypic profile shows two distinct groups of CVID patients

**Figure 1:**
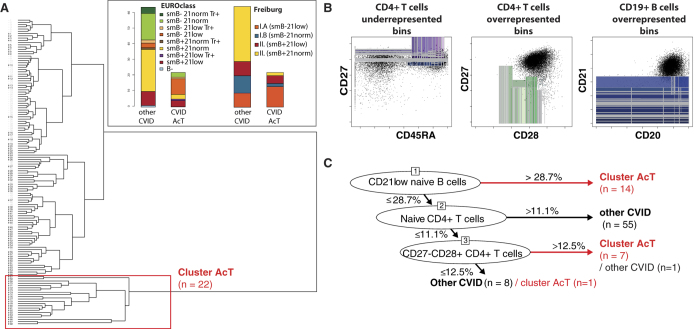
Unsupervised clustering of the phenotypic profiles of lymphocytes in CVID patients. Hierarchical tree of CVID patients (black) and healthy donors (grey) clustered with respect to B- and CD4+ T-cell phenotypes using a probability binning approach. A distinct cluster of CVID AcT patients (red frame) was formed (**A**). Although the comparison showed a non-random pattern, the composition of the clusters did not follow the EUROclass nor Freiburg classifications (inset **A**). To determine the phenotypic hallmarks of the clusters, differentially fed bins in CVID-Act were determined (highlighted as colored rectangles) and projected over a typical phenotype of normal healthy controls (black dots) (**B**). A classification tree was fitted using conventionally gated B-cell and CD4+ T-cell subsets (see gating in Fig. 2) in order to find a simplified definition of CVID- AcT patients (**C**).

To understand the heterogeneity of CVID and in an attempt to delineate groups of patients sharing similar pathophysiology, we performed unsupervised clustering of the phenotypic profiles of lymphocytes in CVID patients. A composite B-lymphocyte and CD4+ T-lymphocyte phenotype probability binning model was constructed based on samples from 48 healthy donors (a training set containing the same number of events from each sample was used). All samples (88 CVID patients and 48 healthy donors) were then compared to the model and clustered with respect to the dissimilarity matrix. A small cluster of CVID patients clearly separated from both healthy donors and the majority of CVID patients was formed (Fig. 1A). The composition of this cluster did not follow the EUROClass, nor Freiburg, nor Rotterdam CVID classification; however, the comparison showed a non-random pattern (p < 0.001), where most of the CD21low patients fell in the cluster and the SmB + CD21norm patients clustered together with healthy controls (Fig. 1A inset).

The profound dissimilarity of the phenotype reflected in the separation of the cluster (25% of CVID patients) suggested that it could be considered a specific subgroup within the broader CVID diagnosis. We propose to call this subset CVID-AcT (Activation, Thrombocytopenia). To justify such phenotype-based stratification for a chronic disease, we sought to investigate whether (a) the patients in this subgroup share clinical signs, (b) the B- and T- cell populations defining the phenotype are stable for a significant period of time and (c) a quantitative correlation between the severity of clinical complications and the defining population can be demonstrated.

### CVID-AcT patients are defined by CD21low B-cell, naive CD4+ T-cell and T-intermediate population frequencies

**Figure 2:**
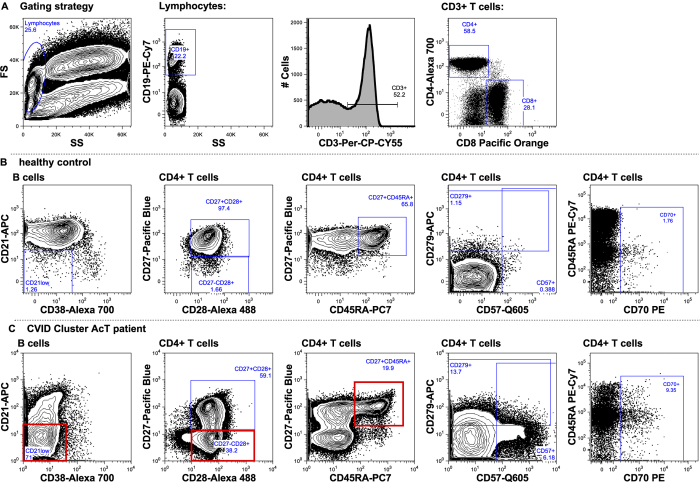
Typical immunophenotype of a CVID-AcT patient. Gating strategy for B and T-cell analysis (**A**), with relevant B and CD4+ T cell subsets displayed in healthy donor (**B**) and in CVID AcT patient (**C**), gates used for simplified definition are highlighted in red.

To identify lymphocyte populations responsible for the immunophenotype dissimilarity between CVID-AcT and the other CVID patients, we created a new probability binning model based on a training set containing the same number of events from both groups in question (as we sought differences within the CVID diagnosis, samples from healthy donors were not used here). We used a multiple testing procedures statistical approach reported previously^18^, and we identified differentially fed bins (i.e., spaces in an 8-dimensional immunophenotype containing significantly different numbers of events between groups) (Fig. 1B). Projection of those bins to conventional dot plots revealed that the hallmark of CVID-AcT within the B-cell phenotype was an inflated CD21low compartment, whereas the T-cell phenotype presented more complex changes: the intermediate CD27−CD28+ compartment was inflated at the expense of reduced naive and central memory compartments, which implied a shift to a more activated phenotype. To simplify the definition of CVID-AcT, we gated commonly assessed B-cell subsets and CD4 T-cell subsets, including the intermediate subset (CD27−CD28+, suggested to be inflated by analysis of overfed bins). We used a classification tree algorithm to select which lymphocyte subsets could be used to define CVID-AcT. We found expanded CD21low B-cells, reduced naive CD4 T-cells and expanded T-intermediate cells as optimal classifiers (all other gated subsets were discarded). A classification tree was able to find cut-offs, based on which 21 of 22 (95%) patients in the CVID-AcT group were correctly discriminated from other CVID patients, while only 1 in 66 of the other CVID patients was misclassified as CVID-AcT (Fig. 1C). The gating of a typical immunophenotype of a CVID-AcT patient is shown in Fig. 2.

### Clinical characteristics of CVID patients

**Figure 3:**
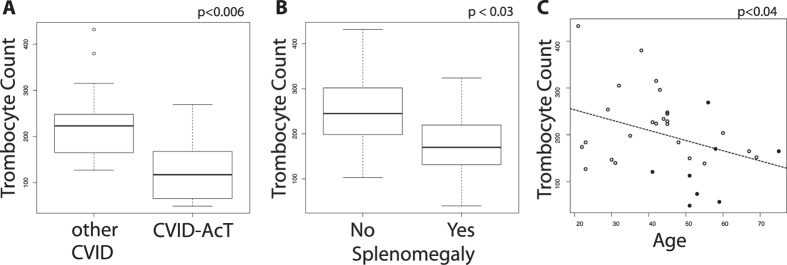
Trombocyte counts are significantly reduced in CVID-AcT patients, patients with splenomegaly and decrease with age. The thrombocyte count is significantly lower in CVID-AcT patients compared to other CVID patients (**A**), as well as in patients with splenomegaly (**B**) and it decreases with age (**C**).

CVID is accompanied by a spectrum of clinical complications (see Table 1). Thrombocytopenia (clinically diagnosed as ITP or Evans syndrome) was found exclusively in patients within delineated phenotype-defined CVID-AcT (8 cases, 36.4%). This prompted us to evaluate the thrombocyte counts (TC) that were in fact reduced in majority of the patients of this cluster (Fig. 3A). Splenomegaly (Fig. 3B) and age (Fig. 3C) were correlated with reduced TC as well. TC was stable in CVID patients for 6–26 month (Supplementary Figure 1). Bronchiectasis and lung fibrosis are frequent and partially overlapping clinical complication in CVID (Supplementary Figure 2). CVID-AcT patients presented with more frequent bronchiectasis (adjusted p < 0.02) and lung fibrosis (adjusted p < 0.0048), and both of the patients with generalized emphysema in our cohort fell into this cluster (Table 1). Fifteen (68%) of the CVID-AcT patients suffered either from lung fibrosis or from bronchiectasis.

### The defining lymphocyte populations show stable frequencies and mutual relationships over the course of the disease

**Figure 4:**
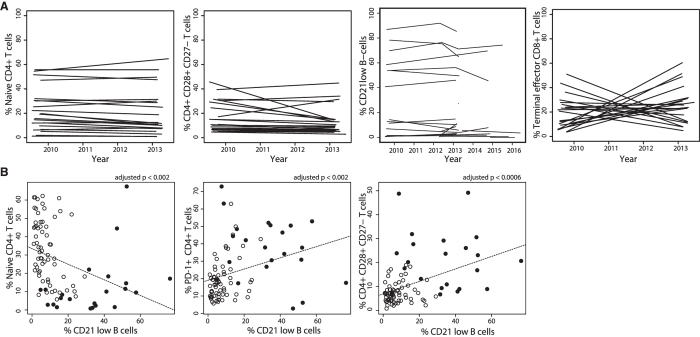
Stability and relationships of defining lymphocyte populations. CVID-AcT patients’ samples contain the same proportion of the defining populations (naïve, intermediate CD28+CD27− CD4+ T-cells, CD21lo B-cells) when measured more than 3 years apart, which is in contrast to the dynamic compartment of terminal effector CD8+ T-cells. The line connecting the two time points is shown to visualize the trends (**A**). Investigation of the mutual relationship of the relevant populations shows orchestrated changes in the patients’ immunophenotypes (**B**).

Once the defining lymphocyte populations were identified, we investigated the stability of these phenotype abnormalities. We previously reported the stability of the B-cell profile in CVID patients over time^18^, we confirmed that CD21low B cells are indeed stable in the present cohort (Fig. 4A). To investigate the stability of the T-cell populations in question, we compared 21 patient samples measured years apart (3.32 to 3.87 years between the two measurements). This comparison revealed an unprecedented stability of the naive CD4+ T-cell population and the CD27−CD28+ intermediate CD4+ T-cell population, which contrasted with the expected variability of the terminal effector CD8+ T-cell compartment (Fig. 4A).

Because the clustering results suggested strong relationships between the populations in the B-cell and CD4+ T-cell compartments, we anticipated that some aberrant regulatory mechanism produced consistent changes in immunophenotype. To determine which phenotypic changes are part of this aberrant mechanism, we investigated the manually gated lymphocyte subsets that were previously reported as abnormal in CVID for their concurrence with populations describing CVID-AcT.

The CD21low B-cells positively correlated with intermediate CD27−CD28+ CD4+ T-cells (adjusted p < 0.0006), and at the same time, the PD-1+ (“exhausted”) CD4+ T-cells correlated with CD21low B-cells (adjusted p < 0.002) (Fig. 4B). The analysis also confirmed a previously described negative correlation of CD21low B-cells with naive CD4+ T-cells (adjusted p < 0.002) and of CD21low B-cells with naive CD8+ T-cells (adjusted p < 0.03)^9^.

### Clinical signs of CVID-AcT patients are correlated with immunosenescence (activation of immune system and loss of naive T-cells)

**Figure 5:**
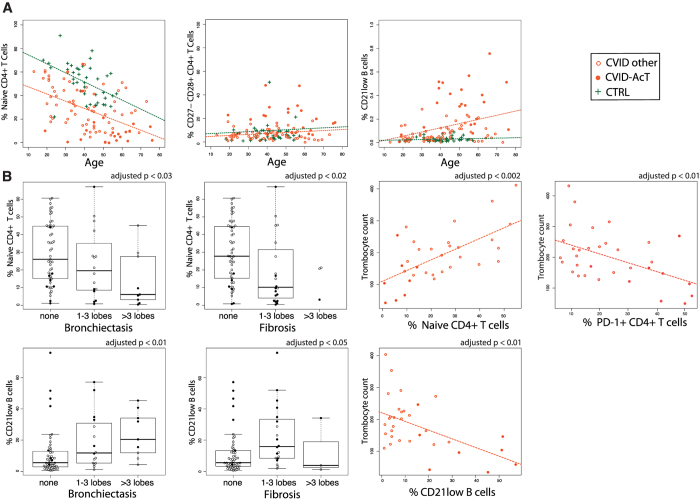
Clinical signs of CVID-AcT patients are correlated with immunosenescence (activation of immune system and loss of naive T-cells). The phenotype of CVID-AcT exhibited signs of immunosenescence in agreement with the higher average age of patients in this cluster. The phenotypic hallmarks of the CVID-AcT patients correlated significantly with age (compared to the T-cell hallmarks both in CVID patients and healthy donors). Additionally, the T-cell phenotype seems to change with age at the same rate in the CVID patients and healthy donors (with no significant difference in the slope), but the CVID patients begin with a decreased number of T-cells compared to the healthy controls (**A**). The phenotypic hallmarks of CVID-AcT correlated significantly (after adjustment for multiple testing) with the severity of their clinical signs (**B**).

While CVID-AcT patients are on average older than other CVID patients (adjusted p < 0.02) (Table 1), the age-based separation of the two clusters is not as clear as the phenotype-based discrimination. Indeed, we observed a different kinetics for the evolution of phenotypic immunosenescence hallmarks (Fig. 5A). The naïve CD4+ T-cells diminished with age in both healthy individuals and CVID patients. However, CVID patients began with lower levels of naïve CD4 T-cells in early adulthood, whereas CD21low B-cells expanded with age only in CVID patients. We investigated the correlation of the immunosenescence-related phenotype of CVID-AcT with the extent of lung fibrosis and bronchiectasis (Fig. 5B). The numbers of CD21low B-cells and naive CD4 T-cells exhibited a significant correlation with the extent of the disorder (1–3 lobes versus >3 lobes involvement). Next, we collected thrombocyte counts (TCs) from 33 patients (11 patients with CVID-AcT). As expected, the TCs were significantly decreased in patients with splenomegaly in their past history (Fig. 3). Interestingly, the TCs had a strong positive correlation with naive CD4+ T-cell numbers and a negative correlation with CD21low B-cells and “exhausted” PD-1+ CD4+ T-cells (Fig. 5B).

Thus far, thrombocytopenia has been mostly associated with splenomegaly; however, the loss of naïve CD4+ T-cells is a stronger predictor than splenomegaly. We performed a step-wise multivariate analysis for modeling the TC using the Akaike information criterion (with all the above-mentioned parameters). The resulting linear model included only 2 predictors of TC: “splenomegaly” and “% Naive CD4+ T-cells”. Although both predictors were able to significantly predict the TC separately (splenomegaly p < 0.02, Naive CD4+ T-cells p < 0.0004), only naive CD4+ T-cells remained a significant predictor in a multivariate model (p < 0.001). While age is an important predictive marker with respect to TC (Fig. 2C), it did not add any significant information to the model which included naive CD4+ T-cell counts. On the other hand, the naive CD4+ T-cell counts significantly improved the model based on age. No clear correlation of other clinical features with age was observed. Thus, we have concluded that it is the “immunophenotypic age” rather than the actual age that characterizes the CVID-AcT patients.

### CVID-AcT is characterized by lymphocyte activation, exhaustion and IL-10 serum elevation

**Figure 6:**
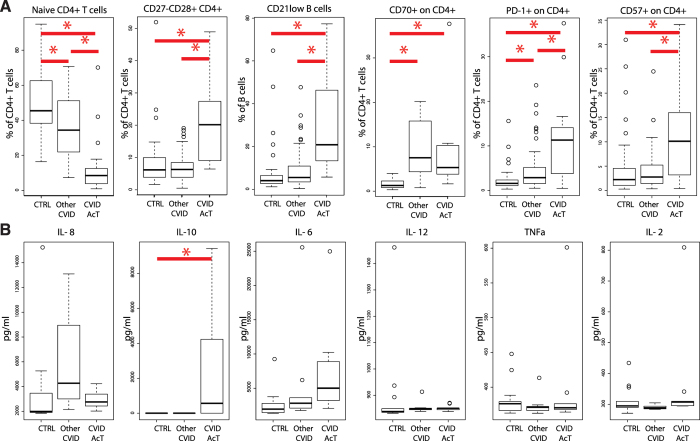
CVID-AcT is characterized by lymphocyte activation, exhaustion and IL-10 serum elevation. Markers of lymphocyte activation, exhaustion and senescence (**A**) and serum cytokine levels (**B**) in healthy controls, other CVID patients and Cluster AcT CVID groups. Asterisk (*) denotes a significant difference between the groups (adjustment p-value using the Benjamini & Yekutieli procedure).

To further investigate whether the abnormal “senescent” immunophenotype of lymphocytes was accompanied by other signs of chronic activation, we investigated CD57 (a marker of senescence or terminal differentiation) and the activation marker CD70 (a ligand of CD27), as well as the exhaustion marker PD-1 (CD279) and plasma cytokine levels. We compared the cytokine profile of CVID-AcT representatives (n = 9) to the other CVID patients (n = 9) and healthy donors (n = 13). While we found strong evidence of exhaustion and senescence in CVID-AcT patients’ CD4+ T-cells (Fig. 6A), we only found increased levels of IL-10 in CVID-AcT compared to the other CVID patients and healthy donors (Fig. 6B). Typical inflammatory cytokines (IL-6, IL-12, TNF-α, IL-2, IFN-γ, soluble CD21, BAFF and G-CSF) were not elevated (Fig. 6B and data not shown).

Because the phenotypic characteristics agreed with the changes described for CMV-positive CVID patients, and the causal effect of IL-10 on thrombocytopenia was previously described^31^, we tested the possibility that the puzzling elevation of IL-10 in otherwise strongly activated phenotypes results from viral IL-10 homolog secretion. An ELISA assay specific for CMV IL-10 and EBV IL-10 showed no differences between CVID-AcT and other CVID samples. All analyzed CVID-AcT patients presented detectable levels of a CMV IL-10 homolog, which was concordant with the presence of a CMV-specific T-cell response (data not shown). CMV persistence was confirmed in 8 out of 9 CVID-AcT patients and in 11 out of 15 other CVID patients by intracellular cytokine staining after CMV peptide stimulation, as described previously^32^,^33^.

### Genetic abnormalities in CVID-AcT

We have searched for common genetic abnormalities in CVID-AcT patients. Samples from seven CVID-AcT patients (randomly chosen by sample availability) were subjected to Whole Exome Sequencing. Variations in the TNFRSF13B (TACI) gene were found in three of the CVID-AcT patients, a C104R mutation commonly reported in CVID was found in one case, and a V220A variation (a rare polymorphism) was found in two other patients (Table 2)^34^,^35^. In one patient, a heterozygous activating mutation in the STAT1 gene was found, which was previously described in a child with immune dysregulation, dermatitis and enteropathy^36^. Furthermore, multiple rare variations in genes associated with immune dysregulation were found in CVID-AcT patients (Table 2). Some of these mutations have been predicted to be damaging but have not been described before; some are heterozygous variants in genes described in PID in a homozygous setting. Thus, we can conclude that there is no single gene responsible for the pathology of CVID-AcT patients, but rather several genes unique to each patient contribute to the disease phenotype.

## Discussion

The heterogeneity in clinical presentation of CVID, the relative rarity of the disease and the thus far elusive molecular pathogenesis are factors inhibiting progress in understanding the disease and the development of better therapeutic approaches. In the current study, we have defined an immunophenotypically distinct subset representing 25% of CVID patients who presented with multiple signs of chronic immune system activation and senescence found in CD4+ T-cells, in B-cells and also in serum cytokines. Furthermore, this group of CVID patients suffered from thrombocytopenia, bronchiectasis and pulmonary fibrosis. We propose to delineate this subset as CVID-AcT (Activation, Thrombocytopenia).

CVID-AcT presented with an extremely high proportion of CD21low B-cells (over 28% in the majority of cases), which was much higher than that found in systemic lupus erythematosus (<10%)^37^, Sjögren’s syndrome (<20%)^16^, rheumatoid arthritis (<10%)^15^ or in a CD21low phenotype definition by EUROClass (10% threshold)^5^. However, CVID-AcT patients overlap only partially with those assigned to group Ia of the Freiburg classification^17^ or EUROClass “smB-21low”^5^. Unlike EUROClass and Freiburg classifications, which are based solely on B-cell phenotype, our study proposes combined criteria based on B-cells and CD4+ T-cells. Combination criteria based on subsets of CD4+ T-cells and B-cells were proposed by large DEFI study^10^, where the clinical groups (infection only, lymphoproliferation, autoimmune cytopenia and chronic enteropathy) were formed first and proportion of subset was found different among them and by Giovannetti^8^ *et al*., who stressed the CD4+ Naïve T cells proportion association to clinical features (splenomegaly and overall severity). Interestingly, none of the classification schemes mentioned above^3^,^5^,^6^,^10^ evaluates thrombocytopenia alone, which is the most distinct feature of CVID-AcT (and it overlaps only partially with “autoimmune cytopenia”). Ideally, an international consensus classification incorporating all the findings should be made.

Thus far, an unreported abnormality in CVID patients was the inflated subset of CD4+ CD27− CD28+ “intermediate” T-cells. This subset had been observed before as a small subset in healthy individuals^38^,^39^, unascribed to any pathological condition. Because CD27 downregulation occurs after interaction with its ligand CD70^40^,^41^,^42^, we tested whether the presence of CD4+ CD27−CD28+ T-cells might be a result of prolonged CD70 expression on the lymphocytes of CVID-AcT patients. However, we found higher levels of CD70 on circulating T-cells in all CVID patients. The observed increase in CD70 expression was similar to the increase found in rheumatoid arthritis patients^40^, where it was suggested to not only induce a chronic T-cell response but also eventually compromise the naive T-cell repertoire and permit the progression of autoreactive clones into the memory compartment, thus allowing autoimmunity. Furthermore, studies in mice have shown that chronic CD70-driven costimulation inhibits germinal center formation^41^ and thus might lead to antibody deficiency.

We have documented that the abnormal immunophenotypic pattern of both T-cell and B-cell subsets was remarkably stable over the course of more than three years of follow-up. The changes in B-cell subsets and T-cell subsets were correlated^9^, which suggests that the immune systems of CVID patients reach an abnormal steady state. We can think of this as a “canalization” into a particular phenotype within an epigenetic landscape^43^.

Our approach was to consider the coordinated changes in the immunophenotype of CVID patients and relate these changes to the patients’ clinical presentation. We identified dominant features of the immunophenotype and found them to be consistent with immunosenescence^44^. The CD4+ T lymphocytes of CVID patients appeared to be more senescent than age-matched healthy adults, and CVID-AcT patients were found on the extreme side of this overall trend. As reported by Giovanetti *et al*.^8^ and Vlková *et al*.^9^, naïve CD4 T cells were lower for age in CVID patients, in our cohort the immunosenescence in CVID followed the same rate as seen in healthy individuals (see the slope of the reduction of naïve CD4 T-cells with age), but CVID patients showed an initial “handicap”, which made them look immunologically older than appropriate for their age. Similar immunophenotype shifts to immunosenescence were observed in patients with genetic defects resulting in their failure to control lymphocyte activation (activated PI3K, activated STAT3 or LRBA deficiency, unpublished observation), and immunosenescence is thought to contribute to autoimmunity in rheumatoid arthritis development^44^.

Failure to control lymphocyte activation could also contribute to the clinical conditions found in CVID-AcT: bronchiectasis, lung fibrosis and thrombocytopenia.

Secondary ITP (CVID related) is defined by ASH guidelines as TC below 100 × 10^9^/L^45^. Lower TC in CVID-AcT would fulfill this criterion in only 50% of patients (4/8 patients with TC information). ITP can be a sequel of both impaired platelet production and/or an increased platelet destruction mediated by autoreactive B/T-cells^46^. The spleen is central to phagocytic clearance of opsonized platelets (as reviewed by Lo and Deane^47^). The platelet production was decreased in healthy volunteers receiving rhIL-10^31^, implying that the high levels of IL-10 found in CVID-AcT patients could impair the platelet production. Increased levels of IL-10 in CVID were described previously^48^. However, the high levels of IL-10 observed seemed to be in contradiction to a study that reported decreased IL-10 production in ITP^49^. We speculated that the high levels of IL-10 in CVID AcT patients could have been caused by an IL-10 viral homologue produced by CMV or EBV *in vivo*, but we have found no difference in CVID-AcT compared to the other CVID patients. While the initial trigger of immune activation in CVID remains unknown, Marashi *et al*. suggest CMV infection^50^,^51^. Indeed, the prevalence of CMV was high (89%) in CVID-AcT patients. Thrombocyte sequestration in splenomegaly can also cause ITP (thrombocytopenia is found in patients with splenomegaly in our cohort). However, splenomegaly is a frequent finding in all CVID patients (58% in our cohort), and there was no difference in its incidence in CVID-AcT patients compared to the other CVID patients, suggesting that other factors may also contribute to the development of thrombocytopenia.

Similar to the previous next-generation sequencing study in an unselected cohort of CVID patients^52^, we did not find any common genetic variation that could be responsible for the stable immunophenotype observed in CVID-AcT patients. Every patient in our study had a unique set of several hits in genes responsible for B/T-cell receptor signaling and regulation, non-homologous end joining, V(D)J recombination or other immune system mechanisms.

We propose the following process to be responsible for the CVID-AcT phenotype: activation by a persistent virus, failure to repress that activation (i.e., the IL-10 regulatory system is triggered but is not effective in suppressing the activation), abnormal costimulation (prolonged CD70 expression) or thus far undetected combinations of polymorphisms in activation-regulating genes. These can lead to naïve CD4+ T-cell pool depletion and thus an increased likelihood of promoting autoreactive T-cells to memory stages concurrently with B-cell activation. However, activated B-cells cannot progress through germinal center reactions and fail to produce isotype-switched antibodies. Autoreactive T-cells and/or increased levels of IL-10 impair the survival or production of platelets.

Thus, CVID-AcT patients are an example of CVID where the failure to produce antibodies is only one facet of a complex dysregulation of lymphocyte activation. Our study highlights the association of T-cell immunosenescence with CVID and thrombocytopenia.

## Additional Information

**How to cite this article**: Stuchlý, J. *et al*. Common Variable Immunodeficiency patients with a phenotypic profile of immunosenescence present with thrombocytopenia. *Sci. Rep.* **7**, 39710; doi: 10.1038/srep39710 (2017).

**Publisher's note:** Springer Nature remains neutral with regard to jurisdictional claims in published maps and institutional affiliations.

## Supplementary Material

Supplementary Figures

## Figures and Tables

**Table 1:** Clinical characteristics of the two groups of patients, (*) denotes significantly different variables.

Clinical characteristics	Cluster other CVID	Cluster AcT
Count	66	22
Age	Median 40.5y (Range 13–74)	Median 53.5y (Range 17–76)
Sex	Female 37/Male 27	Female 11/Male 11
Age of onset (*)	Median 21y (Range 1–65)	Median 33y (Range 3–62)
Years on Ig treatment	Median 8y (Range 1–26)	Median 13y (Range 1–27)
Pneumonia on Ig treatment	12 (18.2%)	6 (27.3%)
Diarrhea	17 (25.8%)	6 (27.3%)
intermitent	10 (15.2%)	3 (13.6%)
permanent	7 (10.6%)	3 (13.6%)
Granuloma	4 (6.1%)	1 (4.5%)
Bronchiectasis (*)	13 (19.7%)	12 (54.5%)
1–3 lobes	10 (15.2%)	6 (27.3%)
>3 lobes	3 (4.5%)	6 (27.3%)
Fibrosis (*)	10 (15.2%)	13 (59.1%)
1–3 lobes	8 (12.1%)	12 (54.5%)
>3 lobes	2 (3%)	1 (4.5%)
Emphysema	6 (9.1%)	2 (9.1%)
1–3 lobes	6 (9.1%)	0 (0%)
>3 lobes	0 (0%)	2 (9.1%)
Splenomegaly	28 (42.4%)	13 (59.1%)
Lymphadenopathy	14 (21.2%)	7 (31.8%)
Autoimmune phenomena	16 (24.2%)	9 (40.9%)
ITP or Evans sy (*)	0 (0%)	8 (36.4%)

Unselected patients’ samples were accrued into the study, however, 10 patients were not used in the analysis because of the lack of B-cells (<1% of lymphocytes). Autoimmune phenomena includes any of the following: psoriasis, pernicious anemia, diabetes, vitiligo, celiac-like disease, Crohn disease, ulcerous colitis, autoimmune thyroiditis, vasculitis, AIHA, ITP or Evans syndrome.

**Table 2:** Whole-exome sequencing data in CVID-AcT patients.

Patient	Variants reported in the literature as associated with immune dysregulation	Rare variants predicted as deleterious in genes associated with immune dysregulation
Patient #1 (female)	**STAT1** p.V266I (het. activating)-described by Uzel *et al*.^36^**NLRP3** p.V198M het., **MEFV** p.I591T het. - both variants described in periodic fever syndromes, but also occur in healthy people	**MASP2** p.E93Q het.
		**PTPRC** p.S325R het.
		**IL1A** p.R85Q het.
		**FASN** p. A613T het.
		**TUBB1** p.G109E
		**LIG1** p.R64C het.
Patient #2 (female)		**NCF2** p.H221fs*43 het.
		**RAG1** p.R449K het.
		**RAG2** p.F386L het.
		**FBN1** p.G47S het.
		**EPG5** p.D2208G het.
Patient #3 (male)	**TNSFR13B (TACI)** C104R het. - described in CVID, but also occurs in healthy people	**LRBA** p.1531V het.
		**CFHR5** p.E163fs*35, p.C208R, p.E383* (all het.)
		**PRKDC** p.P695S het. (SIFT: activating)
Patient #4 (male)		**LRRC8A** p.S199L het.
Patient #5 (male)	**TNSFR13B (TACI)** V220A het.	**TYK2** p.R703W het.
	-described in CVID cohorts, but but also occurs in healthy people, rather a polymorphism	**ATM** p.T21A het.
		**TUBB1** p.274M
Patient #6 (female)		**CHD7** p.S103T het.
		**SF3B1** p.R115W het.
		**LAX1** p.C165* het.
		**IL1A** p.R85Q het.
		**FASN** p.R1824W het.
		**NBPF15** p.R1751C het.
Patient #7 (female)	**TNSFR13B (TACI)** V220A het.-described in CVID cohorts, but but also occurs in healthy people, rather a polymorphism	**TLR1** p.H720P het.
		**UNC13D** splice site loss het.
		**DSG1** p.I739V het.
		**MX1** p.A151T het.
		**FBN1** p.T2520M het.
		**LIG1** p.R64C het.

Rare variants in genes described in immunodeficiency are those with a frequency of less than 1% in population databases (1000 Genomes, NHLBI ESP) and less than 3% in our in-house database of exome data, predicted as damaging by SIFT or PolyPhen-2.
