# Erratum: Common Variable Immunodeficiency patients with a phenotypic profile of immunosenescence present with thrombocytopenia

**DOI:** 10.1038/srep42569

**Published:** 2017-02-27

**Authors:** Jan Stuchlý, Veronika Kanderová, Marcela Vlková, Ivana Heřmanová, Lucie Slámová, Ondřej Pelák, Eli Taraldsrud, Dalibor Jílek, Pavlína Králíčková, Børre Fevang, Marie Trková, Ondřej Hrušák, Eva Froňková, Anna Šedivá, Jiří Litzman, Tomáš Kalina

Scientific Reports 7: Article number: 39710; 10.1038/srep39710 published online: 01 05 2017; updated: 02 27 2017.

The original version of this Article contained errors in the Abstract.

“The degree of severity of these symptoms corresponded to more deviation from normal levels with respect to CD21low B-cells, naïve CD4+ and CD27− CD28+ over three years. Moreover, th-cells”.

now reads:

“The degree of severity of these symptoms corresponded to more deviation from normal levels with respect to CD21low B-cells, naïve CD4+ and CD27− CD28+ CD4+ T-cells”.

These errors have now been corrected in the PDF and HTML versions of the Article.

