# Supplementary material for: Common Variable Immunodeficiency patients with a phenotypic profile of immunosenescence present with thrombocytopenia
Source: Sci Rep. 2017 Jan 5;7:39710. doi: 10.1038/srep39710 (PMC5214528; doi:10.1038/srep39710)
Supplement: Supplementary Figures [file srep39710-s1.pdf]

Common Variable Immunodeficiency patients with a phenotypic profile of immunosenescence present with thrombocytopenia

Jan Stuchlý[1,2], Veronika Kanderová[1,2], Marcela Vlková[3,4] , Ivana Heřmanová[1,2], Lucie Slámová[1,2], Ondřej Pelák[1,2], Eli Taraldsrud[5], Dalibor Jílek[6], Pavlína Králíčková[7], Børre Fevang[8,9], Marie Trková[10], Ondřej Hrušák[1,2], Eva Froňková[1,2], Anna Šedivá[11], Jiří Litzman[3,4], Tomáš Kalina *[1,2]

1, Department of Paediatric Haematology and Oncology, Second Faculty of Medicine, Charles University and University Hospital Motol, Prague, Czech Republic

2, CLIP - Childhood Leukemia Investigation Prague

3, Department of Clinical Immunology and Allergology, St. Anne's University Hospital in Brno, Czech Republic

4, Faculty of Medicine, Masaryk University, Brno, Czech Republic

5, Department of Immunology, Institute for Cancer Research, Oslo University Hospital Radiumhospitalet, Oslo, Norway

6, Centre of Immunology and Microbiology, Regional Institute of Public Health, Usti nad Labem, Czech Republic

7, Institute of Clinical Immunology and Allergology, University Hospital, Hradec Kralove, Czech Republic

8, Research Institute of Internal Medicine, Clinic of Cancer, Inflammation and Transplantation, Oslo University Hospital.

9, Section of Clinical Immunology and Infectious Diseases, Clinic of Cancer, Inflammation and Transplantation, Oslo University

10, Gennet, Prague, Czech Republic

11, Department of Immunology, 2nd Faculty ofMedicine, Charles University and University Hospital Motol, Prague, Czech Republic

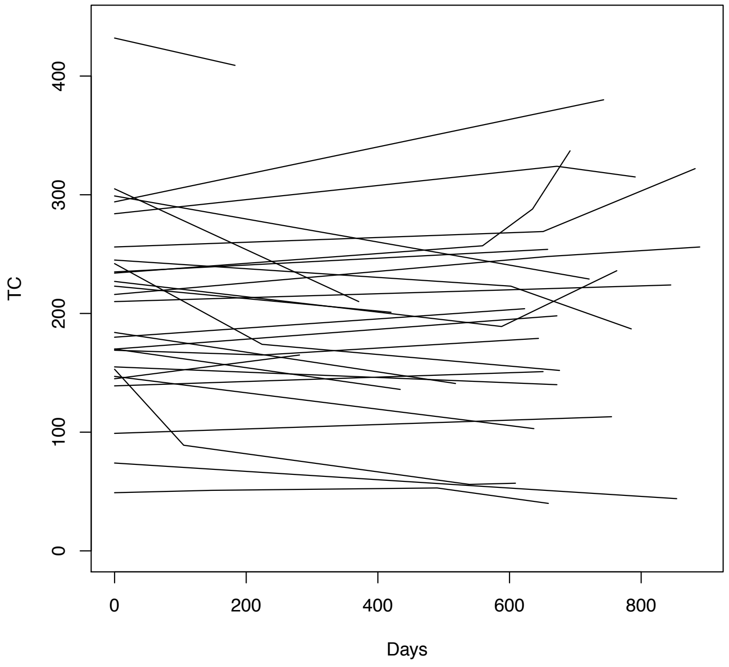

Supplementary Figure 1
Trombocyte Count (TC) is stable over period of 6-26 month in 25 patients with CVID

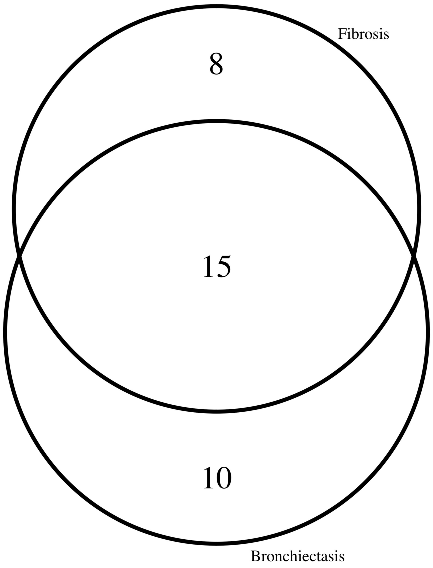

Supplementary Figure 2
Presence of bronchiectasis and fibrosis significantly (fisher exact test $p<0.0005$) overlaps in 15 of 33 CVID patients with lung complications.
